# Intensified Upadacitinib Dosing for Adolescent Patients with Acute Severe Ulcerative Colitis

**DOI:** 10.3390/children12040401

**Published:** 2025-03-22

**Authors:** Perseus V. Patel, Martina Rigmaiden, Alka Goyal, Rachel Bensen, Dorsey Bass, Jonathan Moses, Michael J. Rosen, Ruben J. Colman

**Affiliations:** Division of Pediatric Gastroenterology, Department of Pediatrics, Stanford Medicine Children’s Health, Center for IBD and Celiac Disease, Stanford University School of Medicine, Palo Alto, CA 94304, USA; patelpe@stanford.edu (P.V.P.);

**Keywords:** children, inflammatory bowel disease, targeted small-molecule therapy, janus-kinase inhibitor, pediatric

## Abstract

**Background/Objectives**: In adolescent patients with ulcerative colitis refractory to anti-tumor necrosis factor (TNF) therapy, episodes of acute severe ulcerative colitis (ASUC) require hospitalization or surgery. Upadacitinib can be a potential colectomy-sparing agent in adult ASUC patients receiving intensified dosing. **Methods**: This case series evaluates clinical outcomes of intensified rescue upadacitinib dosing in adolescent patients with ASUC. We included adolescents admitted with anti-TNF refractory ASUC treated with 30 mg twice daily upadacitinib. The primary outcome was the proportion of patients who remained colectomy-free at the most recent follow-up. **Results**: Five patients (aged 14–18) exhibited varying responses to upadacitinib; 2 responded rapidly, while 3 had partial response. All the patients remained on upadacitinib and were colectomy-free during follow-up (55–203 days). Three (60%) ultimately received dual advanced therapy with ustekinumab and upadacitinib. At most recent follow-up, 60% were in clinical/biochemical remission without corticosteroids. **Conclusions**: In select cases, intensified upadacitinib may be a potential colectomy-sparing option for adolescent ASUC patients refractory to anti-TNF therapy.

## 1. Introduction

Intravenous corticosteroids and infliximab are mainstays of therapy in acute severe ulcerative colitis (ASUC) [1]. Upadacitinib (UPA), a selective Janus kinase-1 inhibitor (JAKi), was recently approved for adults with ulcerative colitis (UC) and Crohn’s disease (CD) [2]. Given its rapid onset of action, short half-life, and independence from monoclonal antibody clearance factors that are associated with colectomy risk, UPA represents an intriguing treatment option for ASUC [3]. Adult ASUC data suggest that UPA may be an effective, colectomy-sparing therapy, including in anti-tumor necrosis factor (TNF) refractory disease [4]. Intensified twice-daily (30 mg BID) dosing is hypothesized to be beneficial in ASUC, as drug absorption occurs primarily within 6 h of administration [4]. In a retrospective series of adult ASUC patients, most of whom received intensified UPA induction, 76% remained colectomy-free at 3 months [4].

UPA is not Food and Drug Administration (FDA), or European Medical Association (EMA) approved for use in pediatric inflammatory bowel disease. Data on the use of UPA in adolescent patients with UC are sparce, primarily limited to non-ASUC and once daily dosing [5,6]. As 15% of pediatric and adolescent patients experience severe flares, and up to 30% are anti-TNF non-responders, real-world evidence for safe and effective treatment options in ASUC beyond colectomy is needed [1]. This study presents our experience with intensified rescue UPA dosing in adolescent patients with ASUC.

## 2. Materials and Methods

This retrospective single-center series includes adolescent patients (10–19 years old) hospitalized for ASUC (pediatric UC activity index (PUCAI) ≥ 65 points) and treated with intensified UPA, defined as 30 mg twice daily. Patients meeting the above criteria were consecutively included from 1 July 2023, to 30 June 2024. The patients may have been previously exposed to advanced therapies. We excluded patients with CD, and those without ASUC who only received standard UPA induction dosing (45 mg daily), as we were underpowered to draw comparisons. During the observation period, no patients with ASUC meeting criteria for this study were treated differently.

Under an IRB-approved protocol, we collected data including demographics, disease characteristics, hospitalization duration, inpatient ASUC medications, PUCAI, serum markers (hemoglobin, C-reactive protein, albumin), and calprotectin. The baseline (inclusion) time point was considered the day that they initiated UPA at the intensified dose. We collected post-discharge data including symptoms, UC treatments, and need for re-admission, therapy change, or colectomy. Follow-up visit frequency was based on the treating physician’s discretion. The primary outcome was incidence of colectomy by the time of the most recent follow-up visit. Secondary outcomes included clinical remission off corticosteroids at time of assessment [7], and UPA discontinuation at most recent follow-up. We evaluated adverse events including dyslipidemia, thrombosis, acne, or serious infections including varicella zoster. Our institutional clinical protocol on the use of intensified dosing is adapted from the University of Michigan [8].

### 2.1. Case Summaries

#### 2.1.1. Patient 1

Patient 1 was an 18-year-old male with steroid-dependent UC (E2, S1) refractory to 5-aminosalicylate and infliximab (primary non-response). Upon ASUC admission (Table 1), he was initiated on IV methylprednisolone and UPA 45 mg daily, with partial response (PUCAI = 45) on day 6. Given minimal improvement, the surgery team was consulted to discuss colectomy. In shared decision-making with patient and family, the patient preferred trial of additional medical therapies. Subsequently, we trialed intensified UPA for 5 days, with minimal additional response. He then received intra-arterial prednisolone to target the left colon (research protocol [9]) and concurrent ustekinumab (UST) induction. His PUCAI improved to 25, and he was discharged. At most recent follow-up, 5 months post-discharge, he was in biochemical remission (normalized PUCAI, C-reactive protein and calprotectin) on UST and UPA, and off corticosteroids for 16 weeks.

#### 2.1.2. Patient 2

Patient 2 was an 18-year-old male with UC (E2, S1), with primary non-response to infliximab. He had recently started UPA 45 mg daily for 10 days prior to admission. The surgery team was consulted to discuss colectomy; the family preferred additional medical therapy, and the patient started intensified UPA. On day 4, the patient was given partial response, also initiated on IV methylprednisolone, oral vancomycin and doxycycline. On this combination, PUCAI improved to 45 (Table 2). He was induced on UST, and ultimately discharged on UPA 45 mg daily, UST, and prednisone. He completed a 7-week prednisone taper, and at the most recent follow-up, 3 months post-discharge, he was in clinical remission on UST and UPA, and off corticosteroids for 4 weeks.

#### 2.1.3. Patient 3

Patient 3 was a 15-year-old female with UC (E3, S1) whose infliximab was discontinued after an anaphylactic reaction. Her UC symptoms worsened within a week of her last infusion, and she was admitted for ASUC. Following a surgical consult, she initiated UPA 30 mg BID. Within 4 days, she improved and was transitioned to standard UPA induction. Post-discharge, she was unable to transition to maintenance dosing or wean off steroids due to persistent moderate disease. Four months later, she was re-admitted with moderate-severe disease (PUCAI = 60) and was induced on UST and IV methylprednisone. At the most recent follow-up, 9 months post-discharge, she had moderate disease activity on ongoing dual therapy (UPA and UST) and corticosteroids.

#### 2.1.4. Patient 4

Patient 4 was a 16-year-old female with UC (E4, S1) whose disease was refractory to 5-aminosalicylate and adalimumab (primary non-response) and was undergoing induction with UPA 45 mg. Shortly after starting UPA, she was admitted for ASUC, adenovirus, and respiratory metapneumovirus infections. She was started on IV methylprednisolone, and UPA was held. On hospital day 5, she restarted UPA 45 mg daily and oral vancomycin for anti-inflammatory treatment. A repeat colonoscopy on day 8 showed Mayo 2 disease, and her PUCAI remained severe (65). Colectomy was discussed, but the patient preferred additional medical therapy. She was escalated to intensified UPA for 4 days. Her PUCAI improved (30), and serum inflammatory markers normalized. She was discharged on UPA 45 mg, prednisone and vancomycin. She had moderate disease activity at last follow-up (6 months post-discharge) and continues UPA 45 mg daily.

#### 2.1.5. Patient 5

Patient 5 was a 14-year-old male with UC (E4, S1) whose disease had primary non-response to infliximab. After admission, he was started on IV methylprednisolone and UPA 30 mg BID. His PUCAI decreased to 35 by day 4, and he was discharged on a 4-week prednisone taper and UPA 45 mg daily. At his most recent visit, 2 months post-discharge, he is in clinical remission on UPA, and off corticosteroids for 6 weeks.

## 3. Results

Five adolescents (14–18 years-old) were included in this study. All were anti-TNF exposed, and two were undergoing standard-dose UPA induction at the time of their ASUC hospitalization (Table 1).

All patients were treated with intravenous methylprednisolone (1 mg/kg; maximum 40 mg daily), in accordance with consensus guidelines [10], and offered colectomy as a therapeutic option. Intravenous corticosteroids have been shown to reduce mortality in ASUC, and remain a cornerstone of inpatient treatment [11]. Patients who opted for additional medical therapy were treated for 3–5 days with intensified UPA dosing and subsequently transitioned to the standard induction dose. Two patients started advanced combination therapy with ustekinumab during their hospitalization, and one of those also received gut-directed intra-arterial methylprednisolone [9], given persistent moderate disease activity following intensified UPA doses (Table 2). Of note, both patients were undergoing UPA induction prior to admission. All the patients were discharged on standard UPA induction, with hospitalizations lasting 8–19 days (Table 2).

There were no serious adverse events. Per the guidelines, all the patients received enoxaparin thromboprophylaxis while hospitalized [10,12]. Two patients developed cystic acne on UPA 45 mg daily. They were successfully treated with topical therapy, and acne has not recurred.

At the time of the last outpatient follow-up [range: 55–203 days], all the patients were colectomy-free on UPA and 60% were in remission (Table 3). Three patients completed 8-week inductions and successfully de-escalated to maintenance dosing. Two patients continued to have moderate disease and were re-discussing colectomy as a potential therapeutic option.

## 4. Discussion

To our knowledge, this is the first case series describing the use of intensified UPA induction in anti-TNF-exposed adolescent patients with ASUC. Medical therapy options for these patients are limited given their severe phenotype. The most recent pediatric ASUC guidelines preceded the approval of UPA [10]. While the initial management of these cases was aligned with guideline recommendations, the severity of their disease processes necessitated treatment escalation beyond the scope of previous guidelines. Our results suggest a role for UPA as a potential therapy to avert emergent colectomy in otherwise severe, refractory disease.

There were no serious adverse events. Two patients developed acne, which resolved with topical treatment. Importantly, even at the intensified dose, there were no thrombotic events when using prophylactic enoxaparin, and no infectious complications despite patients being on multiple immunosuppressants.

All the patients clinically improved on UPA, although to varying degrees. Two patients (40%) had dramatic improvement within 5 days. This aligns with adult studies demonstrating clinical response within day 3 of induction [3]. Others had a partial response to the combination of corticosteroids and UPA, including one patient who received intra-arterial steroids targeted to the colon as per a non-related research protocol aimed at limiting systemic corticosteroid-exposure [9]. For these patients, the improvement from severe to moderate disease activity was essential as it allowed for shared decision-making with families regarding pursuing advanced combination therapy versus colectomy as the next step in care. In discussion with families, we opted to use UST as advanced combination therapy with UPA. The combination of JAKi and anti-IL-12/23 appears effective in refractory pediatric and adolescent cases [5]. UST offers a complementary mechanism of action, and has shown early symptomatic response in patients with UC, which made it a more attractive option than vedolizumab in this clinical situation [13].

Ultimately, 60% of patients required advanced dual therapy, which aligns with previously reported pediatric and adolescent data in which 45% of patients required similar combination therapy [5]. It remains unclear if this is due to disease severity, or lack of durability of JAKi monotherapy in adolescent patients. Furthermore, our patients who required dual therapy did not initially respond to standard UPA induction, suggesting that dose escalation may not capture remission. Our results are limited by the small sample size that may influence selection bias [14], as our cohort may not be representative of the larger adolescent ASUC population. Additionally, we did not have endoscopic follow-up and no comparator to standardized UPA dosing or corticosteroids alone. Larger pediatric and adolescent prospective trials are needed to validate safety and effectiveness, as well as maintenance of longer-term corticosteroid-free remission. While we did not detect serious adverse events, our study is underpowered to evaluate safety in this population. Furthermore, as with any retrospective real-world study, multiple interventions were undertaken simultaneously during clinical care, making it difficult to ascertain causality. In conclusion, we hypothesize that due to its rapid mechanism of action, and small-molecule pharmacokinetics that are not influenced by monoclonal antibody clearance-related factors [15], UPA may be an option in anti-TNF-exposed adolescent patients with ASUC. Intensified UPA dosing may have helped avert emergent colectomy in patients with no other medical options. As previously seen with tofacitinib, where intensified induction dosing was protective against early colectomy in ASUC [16], further studies will be required to compare efficacy of intensified versus standard induction dosing in ASUC. With 60% of patients with previously severe, refractory disease now in remission off corticosteroids, future work is needed to elucidate UPA’s role as maintenance monotherapy or as a bridge to advanced combination therapy in adolescent patients with ASUC.

## Figures and Tables

**Table 1:** Demographics and baseline clinical characteristics.

Patient Characteristics					Baseline Assessment						
Patient ID	Gender	Age at Diagnosis	Paris Classification	Previous UC Therapies	Age	Weight (kg)	UC Therapy Regimen Prior to Admission	Steroid Use Prior to Admission	CRP (mg/dL)	Albumin (mg/dL)	Admission PUCAI
1	M	17	E2 S1	* 5-ASA	18	75.8	IFX 800 mg every 4 weeks	No	2.0	3.5	70
2	M	18	E2 S1	^Δ^ IFX	18	45.9	UPA 45 mg daily	No	0.9	4.0	65
3	F	14	E3 S1	IFX	15	44.1	IFX 400 mg × 2	Yes	15.3	2.8	65
4	F	16	E4 S1	^♦^ ADA	16	55	UPA 45 mg daily **	Yes	3.9	3.2	65
5	M	14	E4 S1	IFX	14	40	IFX 600 mg every 4 weeks Metronidazole 250 mg thrice daily Vancomycin 500 mg thrice daily	Yes	<0.3	3.9	65

Demographics and clinical characteristics of patients initiated on intensified induction of upadacitinib (30 mg twice daily). Baseline assessment represents the start of intensified upadacitinib induction dosing. * 5-ASA: oral 5-aminosalicylate. ^Δ^ IFX: infliximab. ^♦^ ADA: adalimumab. ** patient only received 1 dose at home prior to hospitalization.

**Table 2:** Clinical assessments following intensified induction and at hospital discharge.

	End of Intensified Upadacitinib (UPA) Induction					Hospital Discharge				
Patient	Length of Intensified Induction (Days)	^#^ PUCAI	CRP (mg/dL)	Albumin (mg/dL)	Concomitant Therapies	Length of Stay (Days)	CRP (mg/dL)	Albumin (mg/dL)	Concomitant Therapies	PUCAI
1	5	45	1.2	3.6	^Δ^ UST 390 mg IV prednisone 40 mg daily * IA-steroids	17	2	4.5	UPA 30 mg daily ^♦^ Pred 40 mg daily ^^^ Quad ABX	25
2	5	45	0.7	3.6	IV prednisone 40 mg daily Doxycycline 100 mg, twice daily Vancomycin 250 mg, 4 times daily	18	0.7	4.5	UPA 45 mg daily UST 390 mg Pred 35 mg daily	35
3	3	55	1.7	3.2	IV prednisone 40 mg daily	10	0.6	3.4	UPA 45 mg daily Pred 15 mg daily	45
4	5	30	<0.3	2.7	IV prednisone 40 mg daily	14	<0.3	2.6	UPA 45 mg daily Pred 20 mg daily	25
5	4	35	-	3.7	IV prednisone 40 mg daily	8	-	-	UPA 45 mg daily Pred 40 mg daily	35

Clinical and biochemical makers for each patient at the end of the intensified UPA induction, and at the time of discharge. Clinical improvement was determined based upon documentation from the treating physician. Concomitant therapies include all ulcerative colitis treatments given during the intensified. induction phase (column 6) or following intensified induction and up to hospital discharge (column 10). ^Δ^ UST: Ustekinumab weight-based intravenous induction dose. * IA-steroids: single intra-arterial steroid infusion (600 mg methylprednisolone) targeted to the colon. ^♦^ Pred: oral prednisone. ^^^ Quad Abx: quadruple oral antibiotics (vancomycin, metronidazole, doxycycline, amoxicillin). ^#^ PUCAI: pediatric ulcerative colitis activity index.

**Table 3:** Clinical assessment at most recent follow-up.

Patient ID	Ulcerative Colitis Therapies	Time Since Discharge	^#^ PUCAI	Physician Global Assessment	Steroid Use	CRP (mg/dL)	Albumin (g/dL)	Days on Upadacitinib	Adverse Events
1	^Δ^ UST 90 mg every 4 weeks ^♦^ UPA 30 mg daily	5 months	10	Quiescent	No	<0.3	4.5	151	Acne *
2	UST 90 mg every 4 weeks UPA 30 mg daily	3 months	10	Quiescent	No	0.7	4.3	89	None
3	UST 90 mg every 4 weeks UPA 45 mg daily	9 months	45	Moderate	Yes	<0.3	4.4	203	Acne *
4	UPA 45 mg daily	6 months	55	Moderate	No	0.4	4	180	None
5	UPA 30 mg daily	2 months	5	Quiescent	No	<0.3	4.5	55	None

Disease activity and treatment at the time of last known follow-up. ^Δ^ UST: Ustekinumab. ^♦^ UPA: Upadacitinib. * Both patients referred to pediatric dermatology and acne resolved with topical treatments. ^#^ PUCAI: pediatric ulcerative colitis activity index.

## Data Availability

The original contributions presented in this study are included in the article. Further inquiries can be directed to the corresponding author.
